# Minor impact of anastomotic leakage in anterior resection for rectal cancer on long-term male urinary and sexual function

**DOI:** 10.1007/s00384-024-04626-7

**Published:** 2024-04-09

**Authors:** Martin Rutegård, Henrik Jutesten, Pamela Buchwald, Eva Angenete, Marie-Louise Lydrup

**Affiliations:** 1 Department of Surgical and Perioperative Sciences, Surgery, Umeå University, Umeå, Sweden; 2 Wallenberg Centre for Molecular Medicine, Umeå University, Umeå, Sweden; 3 Department of Surgery, Institution for Clinical Sciences Malmö, Lund University, Skåne University Hospital Malmö, Carl-Bertil Laurells Gata 9, 205 02 Malmö, Sweden; 4 Department of Surgery, SSORG - Scandinavian Surgical Outcomes Research Group, Institute of Clinical Sciences, Sahlgrenska Academy, University of Gothenburg, Gothenburg, Sweden; 5 Department of Surgery, Sahlgrenska University Hospital/Östra, Region Västra Götaland, Gothenburg, Sweden

**Keywords:** Anastomotic leakage, Rectal cancer, Urinary and sexual function

## Abstract

**Purpose:**

Anastomotic leakage after anterior resection for rectal cancer induces bowel dysfunction, but the influence on urinary and sexual function is largely unknown. This cross-sectional cohort study evaluated long-term effect of anastomotic leakage on urinary and sexual function in male patients.

**Methods:**

Patients operated with anterior resection for rectal cancer in 15 Swedish hospitals 2007–2013 were identified. Anastomotic leakage and other clinical variables were retrieved from the Swedish Colorectal Cancer Registry and medical records. Urinary and sexual dysfunction were evaluated at 4 to 11 years after surgery using the International Prostate Symptom Score, International Index of Erectile Function, and European Organization for the Research and Treatment of Cancer Quality of Life Questionnaire CR29. The effect of anastomotic leakage on average scores of urinary and sexual dysfunction was evaluated as a primary outcome, and the single items permanent urinary catheter and sexual inactivity as secondary outcomes. The association of anastomotic leakage and functional outcomes was analyzed using regression models with adjustment for confounders.

**Results:**

After a median follow-up of 84 months (interquartile range: 67–110), 379 out of 864 eligible men were included. Fifty-nine (16%) patients had anastomotic leakage. Urinary incontinence was more common in the leakage group, with an adjusted mean score difference measured by EORTC QLQ ColoRectal–29 of 8.69 (95% confidence interval: 0.72–16.67). The higher risks of urinary frequency, permanent urinary catheter, and sexual inactivity did not reach significance.

**Conclusion:**

Anastomotic leakage after anterior resection had a minor negative impact on urinary and sexual function in men.

**Supplementary Information:**

The online version contains supplementary material available at 10.1007/s00384-024-04626-7.

## Introduction

For mid and high rectal cancers, a sphincter-saving anterior resection is often feasible, aimed at preserving bowel continuity [1]. A colorectal anastomosis has a 4–16% risk of anastomotic leakage (AL), with a subsequent high risk of failure to maintain bowel continuity [2]. Additionally, AL may have a negative impact on oncological outcomes, with an increased risk of locoregional recurrence [3–5]. The functional outcome after anterior resection (AR) can be poor with persistent urinary, sexual, and bowel dysfunction, along with a corresponding impact on quality of life [6–10]. A negative effect of AL on bowel function has been reported [11–15] but few studies have focused on long-term urinary and sexual function after AL, with conflicting results [13, 15–17]. Hypothetically, AL might cause neural and/or organ damage leading to dysfunction, either through chronic inflammation or treatment-related injury incurred by drains or reoperations.

The primary aim of this study was to evaluate the long-term effect on urinary function of AL after AR for rectal cancer. A secondary aim was to explore the effect of AL on the risk of urinary catheter permanence and sexual function, including sexual inactivity. Our hypothesis was that AL results in urinary and sexual dysfunction in the long-term perspective.

## Materials and methods

### Study population

Male patients operated with AR for rectal cancer at 15 hospitals in the Northern, Western, and Southern healthcare regions of Sweden between 2007 and 2013 were included in this retrospective cross-sectional multicenter cohort study. The original patient cohort, comprising both men and women, has previously been used to study the influence of non-steroidal anti-inflammatory drug intake on AL [18], as well as the impact of AL on low anterior resection syndrome (LARS) [14]. Clinical variables including the exposure of AL were collected from the Swedish Colorectal Cancer Registry (SCRCR) and medical records as previously reported [18]. Medical records were reviewed to validate AL and to identify unregistered ones.

All patients from the original cohort registered as alive in the Swedish population registry were invited to participate by posted letter. Patients with a recorded local recurrence in the SCRCR were excluded. The original intent was to study both men and women. However, the female sample size was smaller (*N* = 280) with a lower AL rate (8.9%). This in conjunction with generally lower response rates to the instruments regarding urinary and sexual function among women resulted in inadequate data for a reliable analysis and thus only men were studied.

### Ethical approval

The regional ethics review board at Umeå University approved the study (Dnr 2017–486-32 M).

### Study exposure: anastomotic leakage

AL was defined as leakage from any staple or suture line or pelvic abscess (with or without radiologically verified leakage) detected within 3–90 days after index surgery in accordance with the definition provided by the International Study Group of Rectal Cancer (ISREC) [19]. The diagnosis was made using radiology (computerized tomography, rectal contrast study, or magnetic resonance imaging), endoscopy, or clinical findings (digital examination, drain contents, or operative findings).

### Study outcome: urinary and sexual dysfunction

Urinary and sexual dysfunction were evaluated by responses to a postal questionnaire sent to patients between May and August 2018. The questionnaire included the instruments International Prostate Symptom Score (IPSS), International Index of Erectile Function (IIEF), and European Organization for Research and Treatment of Cancer Quality of Life Questionnaire (EORTC QLQ) ColoRectal cancer module 29 (QLQ–CR29), as well as questions regarding current stoma status and a written consent form. The patients received a postal reminder once within 6 weeks of first dispatch.

The IPSS contains seven questions and patients without symptoms score 0 while maximum score is 35. The IIEF includes 15 questions on 5 different domains (erectile function, intercourse satisfaction, orgasmic function, sexual desire, and overall satisfaction), giving a score from 5 to 25 where a higher score indicates better function. For sexually inactive patients, this summary score was treated as missing. The EORTC QLQ-CR-29 measures functions relevant to patients treated for colorectal cancer and is used as a complement to the general questionnaire EORTC QLQ-C30. The 29 questions in QLQ-CR-29 are used to generate 4 scales, and 19 single items evaluate individual functions or symptoms. Among these, the scale for urinary frequency and the single items urinary incontinence and dysuria were used. The questionnaires are linearly transformed to provide a score from 0 to 100 where a high score corresponds to a high level of symptoms. Scoring and handling of missing data for EORTC QLQ-CR29 were performed according to established guidelines [20, 21].

### Statistical analysis

Baseline characteristics among responders were presented in relation to the exposure anastomotic leakage as frequencies and percentages for categorical variables and in means or medians and standard deviations (SDs) and interquartile ranges (IQRs) for continuous variables. The responders were also compared to the group of eligible non-responders.

**Fig. 1:**
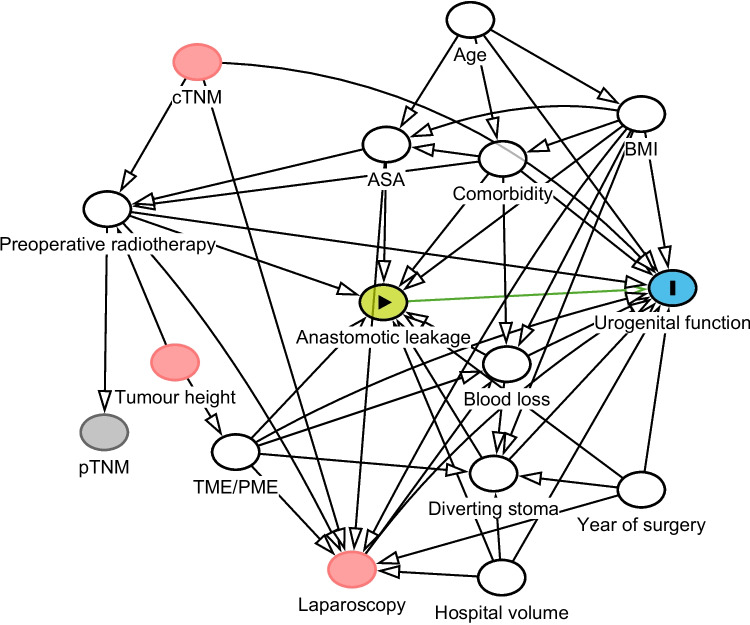
Directed acyclic graph picturing the assumed relationship between different variables potentially involved in the development of male urogenital dysfunction. Anastomotic leakage indicates exposure and urogenital function is outcome. A minimal adjustment set to derive a total effect on the outcome from exposure consisted of variables such as age, body mass index (BMI), comorbidity, American Society of Anesthesiologists’ (ASA) fitness grade, preoperative radiotherapy, total/partial mesorectal excision (TME/PME), blood loss, diverting stoma, hospital volume, and year of surgery

The main aim was to assess the effect of AL on IPSS score and individual urinary items in QLQ-CR29. As secondary outcomes, the risk of urinary catheter permanence as well as sexual dysfunction, including sexual inactivity, assessed by IIEF was analyzed. Linear and logistic regression models were used to estimate the total effect of leakage, adjusting for age (continuous), American Society of Anesthesiologists’ (ASA) fitness grade (I, II, or III), diabetes (yes, or no), cardiovascular disease (yes, or no), body mass index (BMI; continuous), preoperative radiotherapy (yes, or no), blood loss (continuous), type of mesorectal excision (total, or partial), diverting stoma (yes, or no), hospital volume (continuous), and year of surgery (continuous). These covariates were chosen with the help of a directed acyclic graph, where our assumptions about causes and effects involved in the development of urinary and sexual dysfunction are shown (Fig. 1). The amount of missing data for covariates, ranging from 1 to 10%, is presented in Supplementary Table 1. Estimates were presented using coefficients and odds ratios (ORs), as well as 95% confidence intervals (CIs). All analyses used a complete cases approach with the statistical software STATA version 16.1 (StataCorp, TX, USA).

**Table 1:** Baseline characteristics of 379 questionnaire-responding patients operated with anterior resection for rectal cancer, by occurrence of anastomotic leakage

**Baseline characteristics**	**No leakage**	**Leakage**
	***N* = 320**	***N* = 59**
**Age (years)**	66.5 (60.2–71.4)	65.1 (58.4–69.2)
**Body mass index (kg/m**^**2**^**)**	26.0 (23.8–28.1)	25.3 (23.7–28.7)
**ASA fitness grade**		
** I**	83 (26.7%)	23 (40.4%)
** II**	193 (62.1%)	28 (49.1%)
** III**	35 (11.3%)	6 (10.5%)
**Diabetes**		
** No**	296 (92.5%)	56 (94.9%)
** Yes**	24 (7.5%)	3 (5.1%)
**Cardiovascular disease**		
** No**	275 (85.9%)	49 (83.1%)
** Yes**	45 (14.1%)	10 (16.9%)
**Tumor height (cm)**	10.0 (8.0–12.0)	10.0 (8.0–11.0)
**Preoperative radiotherapy**		
** None**	118 (36.9%)	18 (30.5%)
** Short-course 5 × 5 Gy**	152 (47.5%)	29 (49.2%)
Chemoradiotherapy*	48 (15%)	12 (20.3%)
**Annual hospital volume**	19.2 (15.9–36.9)	17.4 (12.9–36.9)
**Year of surgery**	2011 (2009–2012)	2010 (2008–2012)
**Pathological tumor stage**		
** I**	82 (26.6%)	17 (29.3%)
** II**	103 (33.4%)	23 (39.7%)
** III**	114 (37.0%)	16 (27.6%)
** IV**	9 (2.9%)	2 (3.4%)
**Laparoscopic surgery**		
** No**	294 (93.0%)	47 (81.0%)
** Yes**	22 (7.0%)	11 (19.0%)
**Type of mesorectal excision**		
** Partial**	90 (28.7%)	8 (13.8%)
** Total**	224 (71.3%)	50 (86.2%)
**Diverting stoma**		
** No**	47 (14.7%)	6 (10.2%)
** Yes**	273 (85.3%)	53 (89.8%)
**Blood loss (ml)**	450 (250–750)	400 (200–700)

Data are presented as median (IQR) for continuous measures, and *n* (%) for categorical measures. *ASA* American Society of Anesthesiologists

^*^Chemoradiotherapy includes long-course 25–28 × 1.8–2 Gy with capecitabine and short-course 5 × 5 Gy followed by systemic chemotherapy

## Results

**Fig. 2:**
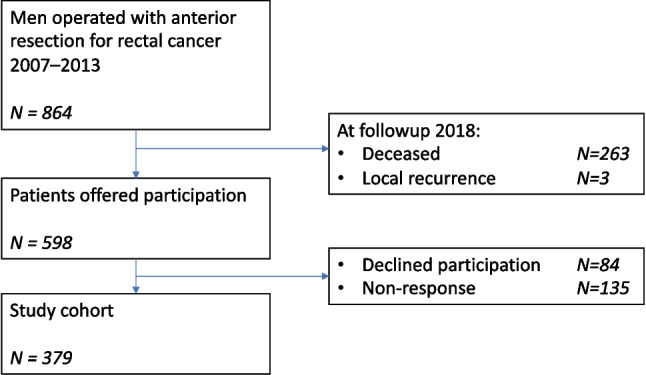
Study flowchart

From 864 male rectal cancer patients operated with AR 2007–2013 in the original cohort, 598 patients were alive at the time of questionnaire dispatch. Of these, 379 (63.4%) patients responded with a median follow-up of 84 months (IQR 67–110) (Fig. 2). Clinical characteristics in responders stratified by AL are presented in Table 1. In total, 59 (15.6%) had AL. Of these leaks, one was grade A, 42 were grade B, and 16 were grade C. In the whole cohort, 318 (83.9%) patients reported bowel continuity compared to 29 (49.1%) in the AL group.

Functional and quality of life outcomes measured by IPSS score, IPSS quality of life, IIEF score, and EORTC QLQ-CR29 urinary symptoms (frequency, incontinence, and dysuria) are shown in Table 2. Unadjusted mean scores for urinary incontinence measured by CR29 were higher in the AL group: 18.7 compared to 13.2 in those without AL. Permanent urinary catheter prevalence (6.8 vs 3.1%) and sexual inactivity (59.3% vs 48.1%) was higher in the AL group compared to the group without AL.

**Table 2:** Outcomes for 379 men operated with anterior resection for rectal cancer and responding to questionnaires on urogenital function, stratified by postoperative anastomotic leakage within 90 days of surgery

**Outcomes**		**No leakage (*N* = 320)**	**Leakage (*N* = 59)**
***Continuous***	***Missing (%)***	***Median (IQR)***	***Median (IQR)***
**IPSS score**	20 (5.3)	6 (2–11)	7 (2–10)
**IPSS quality of life**	15 (4.0)	1 (0–2)	1 (1–2)
**IIEF score**	214 (56.5)	13.5 (7–20)	12 (5–18)
	***Missing (%)***	***Mean (SD)***	***Mean (SD)***
**CR29 urinary frequency**	16 (4.2)	43.9 (23.1)	47.4 (22.3)
**CR29 urinary incontinence**	19 (5.0)	13.2 (25.3)	18.7 (33.9)
**CR29 dysuria**	22 (5.8)	7.1 (21.8)	8.9 (26.6)
***Binary***	***Missing (%)***	***N (%)***	***N (%)***
**Permanent urinary catheter**	21 (6.6)	10 (3.1)	4 (6.8)
**Sexually inactive**	22 (5.8)	154 (48.1)	35 (59.3)

Percentages may not add up due to missing. *IPSS* International Prostate Symptom Score, *IIEF* International Index of Erectile Function, *CR29* colorectal cancer module 29, *IQR* interquartile range

The adjusted analyses are demonstrated in Table 3. A statistically significantly increased urinary incontinence was detected in patients with AL, with a mean score difference of 8.69 (95% CI 0.72–16.67). An increased urinary frequency and a higher rate of permanent urinary catheter in AL patients almost reached statistical significance. The IPSS and IIEF scores were not affected, and no discernible effect of AL on sexual inactivity could be demonstrated in the adjusted analyses.

**Table 3:** Estimation of urinary and sexual function by anastomotic leakage in 379 questionnaire responders, using univariable and multivariable linear and logistic regression. Results are presented with mean score differences and odds ratios (ORs), along with 95% confidence intervals (CIs)

**Outcome**	**Unadjusted**	Adjusted*
*Continuous*	***Coefficient (95% CI)***	***Coefficient (95% CI)***
IPSS score	0.97 (− 1.38–3.32)	1.68 (− 0.83–4.19)
IPSS quality of life	− 0.15 (− 0.52–0.21)	0.00 (− 0.40–0.40)
IIEF score	− 0.91 (− 4.04–2.23)	-0.89 (− 4.33–2.55)
CR29 urinary frequency	3.48 (− 2.98–9.94)	6.12 (− 0.70–12.95)
CR29 urinary incontinence	5.51 (− 2.12–13.14)	8.69 (0.72–16.67)
CR29 dysuria	1.84 (− 4.63–8.31)	2.88 (− 4.05–9.82)
*Binary*	***OR (95% CI)***	***OR (95% CI)***
Permanent urinary catheter	2.22 (0.67–7.36)	4.52 (0.94–21.72)
Sexually inactive	1.43 (0.81–2.54)	1.40 (0.73–2.72)

*IPSS* International Prostate Symptom Score, *IIEF* International Index of Erectile Function, *CR29* colorectal cancer module 29

^*^Adjustment for age, American Society of Anesthesiologists’ fitness grade, diabetes, cardiovascular disease, body mass index, preoperative radiotherapy, blood loss, type of mesorectal excision, diverting stoma, hospital volume, year of surgery

Sexual activity in relation to median age, AL, and stoma status is shown in Supplementary Table 2. In the whole group, 44.3% were sexually active. AL was numerically more common in the sexually inactive group (18.5 vs 13.7%), and a residual stoma was also more prevalent (19.9 vs 9.0%).

### Non-responders and excluded patients

Clinical characteristics in responders compared to non-responders/patients declining participation are outlined in Supplementary Table 3. Non-responders/patients declining participation had higher age at index surgery, more advanced comorbidity, more advanced tumors, and received more neoadjuvant therapy. Of note, the AL rate was similar among non-responders (15.6%).

## Discussion

In this study, a significantly higher risk of urinary incontinence after a median follow-up of 7 years was observed in the leakage group, while IPSS and IIEF scores were not affected. This finding adds further functional morbidity to patients already suffering from other consequences of the leakage. The clinical relevance of urinary incontinence is highlighted in recent publications suggesting an association between urinary incontinence and poor quality of life, especially in men [22] and also an increased risk of death with worsening degrees of incontinence [23, 24].

The relationship of AL after AR and various aspects of functional results have previously been explored with different outcomes, with sparse reporting regarding urogenital dysfunction. The finding of no substantial detrimental effect of AL on long-term urinary function measured by EORTC QLQ-CR29 and IPSS is in line with others. The mean score differences in urinary function between groups, though statistically significant, could be considered only small in nature, as they ranged between 6 and 9 [25]. Mongin et al. reported no difference concerning urinary frequency, incontinence, or dysuria in 21 patients suffering AL compared to a group without AL using CR-29 scores [17]. Similarly, Riss et al. reported no difference in urinary function measured with IPSS in 16 patients with and without AL, while using the International Consultation on Incontinence Questionnaire–Urinary Incontinence Short Form (ICIQ-SF), patients with AL scored significantly higher than the control group [16]. This discrepancy could be explained by the IPSS being a more composite questionnaire on overall urinary function, while ICIQ-SF includes specific questions regarding urinary incontinence. Hain et al. reported urogenital outcome among 23 patients with AL and found significantly increased risk of frequent urination as measured by EORTC-CR29 [13]. These studies corroborate the results in the present study to some degree. In contradiction to our findings, Torrijo et al. [22], with shorter follow-up time than ours (12 months), reported AL as a risk factor for urinary dysfunction measured by IPSS [26]. Our results are also in contrast with Kverneng et al. reporting that AL was associated with a decreased risk for urinary incontinence [15]. However, in that study, urinary function in the AL group at baseline was superior to the group without AL and a non-validated questionnaire was employed. Patients with AL tended to be less sexually active, although not to a statistically significant degree; this is in line with previous findings including no influence on sexual function characterized by IIEF [15, 16].

In this study, 51% of patients experiencing AL still had a stoma, while the corresponding number for patients without AL was 7%. The effect of a permanent stoma on sexual function is difficult to distinguish from the effect of AL. In two recent meta-analyses on quality of life after rectal cancer surgery, worse sexual function was associated with abdominoperineal resection compared to sphincter-saving surgery in men [27, 28]. In abdominoperineal excision of the rectum, more neural damage might occur; thus, the stoma per se might not be the ultimate reason for sexual dysfunction.

The negative impact of AL on urinary and sexual function is minor compared to the substantial impact on bowel function previously reported for this cohort [14]. It is possible that inflammation and fibrosis in the rectal wall influence bowel function more directly compared to a more indirect effect via neural damage on urinary and sexual function. The fact that bowel function was evaluated in patients with bowel continuity, while urinary and sexual function was analyzed in the entire AL group, could also have had an impact on this discrepancy. This study has a long follow-up and several aspects of quality of life have been reported to improve with extended follow-up [29]. This might be explained by symptoms improving but also with a change of expectations, sometimes referred to as response shift [30].

This is one of few studies, with a relatively large AL group, investigating AL effect on male urogenital dysfunction with long-term follow-up. Nevertheless, the still low AL number prohibited subgroup analyses of different leak severity; more importantly, such considerations made it difficult to evaluate female patients as well, as the sample size turned out to be too small for meaningful analysis, despite our initial aim of including both sexes. Moreover, this is a multicenter study, and decreasing selection bias and the use of validated questionnaires make the results comparable to previous research. Furthermore, the medical records of all patients have been reviewed to identify all cases of AL, according to the protocol definition.

A limitation is the response rate of 63.4%, making sampling bias a concern, and there were some differences in the responding compared to the non-responding group, mostly suggesting that the non-responders were frailer with more advanced tumors; this could introduce loss of external validity. The retrospective nature of the study is also a limitation, perhaps contributing to the low response rate. The low number of patients excluded due to cancer recurrence could be explained by misclassifications in SCRCR, though the variables in this registry are reported to be accurate on average in 90% [31]. However, the at most 10% missing data in covariates were regarded as acceptable. Heterogeneity in follow-up time is also a limitation less problematic, as the minimum follow-up time (4 years) probably is beyond the time when further improvement could be expected. A more substantial concern is the lack of knowledge as to what extent urinary and sexual dysfunction were treated. At the time of the study, no structural follow-up programs were in place in Sweden. Treatment of urinary dysfunction was probably limited, while treatment of sexual dysfunction among men after rectal cancer surgery has been more common. There is also a risk of type II error using a relatively small sample of 59 AL patients, limiting the possibility of detecting anything but a large difference in functional outcome. This limitation is further accentuated by the high prevalence of sexual dysfunction also in the non-leakage group. A larger study size might have detected more impact on urinary and sexual dysfunction. In addition, no analysis of the influence of operative treatment of the AL, i.e., further pelvic surgery, has been possible. Speculatively, such procedures could have a further detrimental influence on urinary and sexual function. Future research in this area should be with a prospective design with adequate sample size, a uniform short- and long-term follow-up both using clinical data and patient-reported outcomes.

## Conclusion

This study suggests that AL after AR leads to an increased incidence of urinary incontinence and possibly an increased urinary frequency and permanent catheter use among men 4–11 years after surgery, while no major effect on overall urinary and sexual function was found. This is important information when counselling rectal cancer survivors, enabling awareness of long-term function, especially as urinary incontinence in particular might constitute a major problem for men, as even mortality increases with the degree of incontinence [23, 24].

## Supplementary Information

Below is the link to the electronic supplementary material.

Supplementary file1 (DOCX 14 KB)

Supplementary file2 (DOCX 14 KB)

Supplementary file3 (DOCX 15 KB)

## Data Availability

No datasets were generated or analysed during the current study.

## References

[1] Pachler J, Wille-Jorgensen P (2012) Quality of life after rectal resection for cancer, with or without permanent colostomy. Cochrane Database Syst Rev 12:CD004323 10.1002/14651858.CD004323.pub4 PMC7197443 23235607

[2] Jutesten H, Draus J, Frey J, Neovius G, Lindmark G, Buchwald P, Lydrup ML (2019) High risk of permanent stoma after anastomotic leakage in anterior resection for rectal cancer. Colorectal Dis 21(2):174–182 30411471 10.1111/codi.14469

[3] Borstlap WAA, Westerduin E, Aukema TS, Bemelman WA, Tanis PJ (2017) Dutch Snapshot Research Group. Anastomotic leakage and chronic presacral sinus formation after low anterior resection: results from a large cross-sectional study. Ann Surg 266(5):870–877 28746154 10.1097/SLA.0000000000002429

[4] Bostrom P, Haapamaki MM, Rutegard J, Matthiessen P, Rutegard M (2019) Population-based cohort study of the impact on postoperative mortality of anastomotic leakage after anterior resection for rectal cancer. BJS Open 3(1):106–111 30734021 10.1002/bjs5.50106 PMC6354192

[5] Denost Q, Rouanet P, Faucheron JL et al (2021) Impact of early biochemical diagnosis of anastomotic leakage after rectal cancer surgery: long-term results from GRECCAR 5 trial. Br J Surg 108(6):605–608 33793764 10.1093/bjs/znab003

[6] Emmertsen KJ, Laurberg S (2012) Low anterior resection syndrome score: development and validation of a symptom-based scoring system for bowel dysfunction after low anterior resection for rectal cancer. Ann Surg 255(5):922–928 22504191 10.1097/SLA.0b013e31824f1c21

[7] Bregendahl S, Emmertsen KJ, Lindegaard JC, Laurberg S (2015) Urinary and sexual dysfunction in women after resection with and without preoperative radiotherapy for rectal cancer: a population-based cross-sectional study. Colorectal Dis 17(1):26–37 25156386 10.1111/codi.12758

[8] Sturiale A, Martellucci J, Zurli L et al (2017) Long-term functional follow-up after anterior rectal resection for cancer. Int J Colorectal Dis 32(1):83–88 27695976 10.1007/s00384-016-2659-6

[9] Feddern ML, Emmertsen KJ, Laurberg S (2019) Quality of life with or without sphincter preservation for rectal cancer. Colorectal Dis 21(9):1051–1057 31074098 10.1111/codi.14684

[10] Pieniowski EHA, Palmer GJ, Juul T et al (2019) Low anterior resection syndrome and quality of life after sphincter-sparing rectal cancer surgery: a long-term longitudinal follow-up. Dis Colon Rectum 62(1):14–20 30394987 10.1097/DCR.0000000000001228

[11] Hallbook O, Sjodahl R (1996) Anastomotic leakage and functional outcome after anterior resection of the rectum. Br J Surg 83(1):60–62 8653367 10.1002/bjs.1800830119

[12] Bregendahl S, Emmertsen KJ, Lous J, Laurberg S (2013) Bowel dysfunction after low anterior resection with and without neoadjuvant therapy for rectal cancer: a population-based cross-sectional study. Colorectal Dis 15(9):1130–1139 23581977 10.1111/codi.12244

[13] Hain E, Manceau G, Maggiori L, Mongin C, AlDJ Prost, Panis Y (2017) Bowel dysfunction after anastogmotic leakage in laparoscopic sphincter-saving operative intervention for rectal cancer: a case-matched study in 46 patients using the Low Anterior Resection Score. Surgery 161(4):1028–1039 27894710 10.1016/j.surg.2016.09.037

[14] Jutesten H, Buchwald P, Angenete E, Rutegard M, Lydrup ML (2022) High risk of low anterior resection syndrome in long-term follow-up after anastomotic leakage in anterior resection for rectal cancer. Dis Colon Rectum 65:1264–1273 35482994 10.1097/DCR.0000000000002334

[15] Kverneng Hultberg D, Svensson J, Jutesten H et al (2020) The impact of anastomotic leakage on long-term function after anterior resection for rectal cancer. Dis Colon Rectum 63(5):619–628 32032197 10.1097/DCR.0000000000001613

[16] Riss S, Stremitzer S, Riss K, Mittlbock M, Bergmann M, Stift A (2011) Pelvic organ function and quality of life after anastomotic leakage following rectal cancer surgery. Wien Klin Wochenschr 123(1–2):53–57 21191658 10.1007/s00508-010-1514-y

[17] Mongin C, Maggiori L, Agostini J, Ferron M, Panis Y (2014) Does anastomotic leakage impair functional results and quality of life after laparoscopic sphincter-saving total mesorectal excision for rectal cancer? A case-matched study. Int J Colorectal Dis 29(4):459–467 24477790 10.1007/s00384-014-1833-y

[18] Kverneng Hultberg D, Angenete E, Lydrup ML, Rutegard J, Matthiessen P, Rutegard M (2017) Nonsteroidal anti-inflammatory drugs and the risk of anastomotic leakage after anterior resection for rectal cancer. Eur J Surg Oncol 43(10):1908–1914 28687432 10.1016/j.ejso.2017.06.010

[19] Rahbari NN, Weitz J, Hohenberger W et al (2010) Definition and grading of anastomotic leakage following anterior resection of the rectum: a proposal by the International Study Group of Rectal Cancer. Surgery 147:339–351 20004450 10.1016/j.surg.2009.10.012

[20] Whistance RN, Conroy T, Chie W et al (2009) Clinical and psychometric validation of the EORTC QLQ-CR29 questionnaire module to assess health-related quality of life in patients with colorectal cancer. Eur J Cancer 45(17):3017–3026 19765978 10.1016/j.ejca.2009.08.014

[21] Aaronson NK, Ahmedzai S, Bergman B et al (1993) The European Organization for Research and Treatment of Cancer QLQ-C30: a quality-of-life instrument for use in international clinical trials in oncology. J Natl Cancer Inst 85(5):365–376 8433390 10.1093/jnci/85.5.365

[22] Veronese N, Smith L, Pizzol D et al (2022) Urinary incontinence and quality of life: a longitudinal analysis from the English Longitudinal Study of Ageing. Maturitas 160:11–15 35550703 10.1016/j.maturitas.2022.01.010

[23] John G, Bardini C, Combescure C, Dällenbach P (2016) Urinary incontinence as a predictor of death: a systematic review and meta-analysis. PLoS ONE 11(7):e0158992 27410965 10.1371/journal.pone.0158992 PMC4943733

[24] Åkerla J, Pesonen JS, Pöyhönen A et al (2022) Lower urinary tract symptoms and mortality among Finnish men: the roles of symptom severity and bother. J Urol 207(6):1285–1294 35470712 10.1097/JU.0000000000002450

[25] Musoro JZ, Sodergren SC, Coens C et al (2020) EORTC Quality of Life, Gastro-intestinal Groups. Minimally important differences for interpreting the EORTC QLQ-C30 in patients with advanced colorectal cancer treated with chemotherapy. Colorectal Dis 22(12):2278–2287 32767619 10.1111/codi.15295

[26] Torrijo I, Balciscueta Z, Tabet J, Martín MC, López M (2021) Uribe N Prospective study of urinary function and analysis of risk factors after rectal cancer surgery. Tech Coloproctol 25(6):727–737 33811298 10.1007/s10151-021-02445-4

[27] Maguire B, Clancy C, Connelly TM et al (2022) Quality of life meta-analysis following coloanal anastomosis versus abdominoperineal resection for low rectal cancer. Colorectal Dis 24(7):811–820 35194919 10.1111/codi.16099

[28] Liu XR, Tong Y, Li ZW et al (2023) Do colorectal cancer patients with a postoperative stoma have sexual problems? A pooling up analysis of 2566 patients. Int J Colorectal Dis 38(1):79 36961570 10.1007/s00384-023-04372-2

[29] Ashburn JH, Stocchi L, Kiran RP, Dietz DW, Remzi FH (2013) Consequences of anastomotic leak after restorative proctectomy for cancer: effect on long-term function and quality of life. Dis Colon Rectum 56(3):275–280 23392139 10.1097/DCR.0b013e318277e8a5

[30] Sprangers MA, Schwartz CE (1999) Integrating response shift into health-related quality of life research: a theoretical model. Soc Sci Med 48(11):1507–1515 10400253 10.1016/s0277-9536(99)00045-3

[31] Moberger P, Skoldberg F, Birgisson H (2018) Evaluation of the Swedish Colorectal Cancer Registry: an overview of completeness, timeliness, comparability and validity. Acta Oncol 57(12):1611–1621 30477372 10.1080/0284186X.2018.1529425

