# Supplementary material for: Minor impact of anastomotic leakage in anterior resection for rectal cancer on long-term male urinary and sexual function
Source: Int J Colorectal Dis. 2024 Apr 9;39(1):49. doi: 10.1007/s00384-024-04626-7 (PMC11001750; doi:10.1007/s00384-024-04626-7)
Supplement: Supplementary file 1 — Supplementary file1 (DOCX 14 KB) [file 384_2024_4626_MOESM1_ESM.docx]

**Supplementary Table 1.** Prevalence of missing baseline data among 379 questionnaire responders.

| Variable | Missing data (%) |
| --- | --- |
| American Society of Anesthesiologists’ fitness grade | 2.9 |
| Neoadjuvant therapy | 0.5 |
| Type of mesorectal excision | 1.8 |
| Age | 0.5 |
| Body Mass Index | 9.8 |
| Tumor height | 1.1 |
| Blood loss | 7.1 |
| Hospital volume | 0.5 |
| Year of surgery | 0.5 |
| Pathological tumor stage | 3.4 |
