# Supplementary material for: Minor impact of anastomotic leakage in anterior resection for rectal cancer on long-term male urinary and sexual function
Source: Int J Colorectal Dis. 2024 Apr 9;39(1):49. doi: 10.1007/s00384-024-04626-7 (PMC11001750; doi:10.1007/s00384-024-04626-7)
Supplement: Supplementary file 2 — Supplementary file2 (DOCX 14 KB) [file 384_2024_4626_MOESM2_ESM.docx]

**Supplementary Table 2.** Age, occurrence of anastomotic leakage, and residual stoma by reported sexual activity in 379 patients (22 with missing response).

| Variables | Sexually inactive | Sexually active |
| --- | --- | --- |
|  | **N=189** | **N=168** |
| Age (years) | 67.1 (63.0-72.5) | 63.4 (57.1-69.3) |
| Anastomotic leakage |  |  |
| No | 154 (81.5%) | 145 (86.3%) |
| Yes | 35 (18.5%) | 23 (13.7%) |
| Stoma present |  |  |
| No | 149 (80.1%) | 151 (91.0%) |
| Yes | 37 (19.9%) | 15 (9.0%) |

Data are presented as median (IQR) for continuous measures, and n (%) for categorical measures.
