# Supplementary material for: Minor impact of anastomotic leakage in anterior resection for rectal cancer on long-term male urinary and sexual function
Source: Int J Colorectal Dis. 2024 Apr 9;39(1):49. doi: 10.1007/s00384-024-04626-7 (PMC11001750; doi:10.1007/s00384-024-04626-7)
Supplement: Supplementary file 3 — Supplementary file3 (DOCX 15 KB) [file 384_2024_4626_MOESM3_ESM.docx]

**Supplementary Table 3.** Baseline characteristics of 219 questionnaire non-responding patients operated with anterior resection for rectal cancer, by occurrence of anastomotic leakage.

| Baseline characteristics | No leakage | Leakage |
| --- | --- | --- |
|  | **N=185** | **N=34** |
| Age (years) | 69.1 (62.7-74.7) | 65.8 (57.2-69.9) |
| Body Mass Index (kg/m2) | 25.7 (23.8-27.7) | 25.5 (23.3-29.1) |
| ASA fitness grade |  |  |
| I | 48 (27.1%) | 6 (18.2%) |
| II | 106 (59.9%) | 24 (72.7%) |
| III | 23 (13.0%) | 3 (9.1%) |
| Diabetes |  |  |
| No | 171 (92.4%) | 30 (88.2%) |
| Yes | 14 (7.6%) | 4 (11.8%) |
| Cardiovascular disease |  |  |
| No | 150 (81.5%) | 25 (73.5%) |
| Yes | 34 (18.5%) | 9 (26.5%) |
| Tumor height (cm) | 11.0 (9.0-13.0) | 10.0 (10.0-12.0) |
| Preoperative radiotherapy |  |  |
| No | 87 (47.0%) | 16 (47.1%) |
| Yes | 98 (53.0%) | 18 (52.9%) |
| Annual hospital volume | 17.6 (13.1-36.9) | 18.4 (12.9-43.2) |
| Year of surgery | 2010 (2008-2012) | 2010 (2009-2012) |
| Pathological tumor stage |  |  |
| I | 58 (32.6%) | 7 (20.6%) |
| II | 53 (29.8%) | 14 (41.2%) |
| III | 61 (34.3%) | 13 (38.2%) |
| IV | 6 (3.4%) | 0 (0.0%) |
| Laparoscopic surgery |  |  |
| No | 162 (89.0%) | 27 (79.4%) |
| Yes | 20 (11.0%) | 7 (20.6%) |
| Type of mesorectal excision |  |  |
| Partial | 52 (28.7%) | 2 (6.1%) |
| Total | 129 (71.3%) | 31 (93.9%) |
| Diverting stoma |  |  |
| No | 33 (17.8%) | 3 (8.8%) |
| Yes | 152 (82.2%) | 31 (91.2%) |
| Blood loss (ml) | 425 (200-700) | 600 (250-1200) |

Data are presented as median (IQR) for continuous measures, and n (%) for categorical measures. ASA = American Society of Anesthesiologists’.
